# Insight into *Trichinella britovi* Infection in Pigs: Effect of Various Infectious Doses on Larvae Density and Spatial Larvae Distribution in Carcasses and Comparison of the Detection of Anti-*T. britovi* IgG of Three Different Commercial ELISA Tests and Immunoblot Assay

**DOI:** 10.3390/pathogens11070735

**Published:** 2022-06-28

**Authors:** Michał Gondek, Sylwia Grzelak, Renata Pyz-Łukasik, Przemysław Knysz, Monika Ziomek, Justyna Bień-Kalinowska

**Affiliations:** 1 Department of Food Hygiene of Animal Origin, Faculty of Veterinary Medicine, University of Life Sciences in Lublin, Akademicka 12, 20-033 Lublin, Poland; renata.pyz@up.lublin.pl (R.P.-Ł.); knysz.przemyslaw@gmail.com (P.K.); monika.ziomek@up.lublin.pl (M.Z.); 2 The Witold Stefański Institute of Parasitology, Polish Academy of Sciences, Twarda 51/55, 00-818 Warsaw, Poland; sylwia.grzelak@twarda.pan.pl (S.G.); j.kalinowska@twarda.pan.pl (J.B.-K.)

**Keywords:** pigs, *Trichinella britovi*, intensity of infection, ELISA, Western blot, experimental infection

## Abstract

There is limited information available on the *Trichinella britovi* (*T. britovi*) muscle larvae (ML) distribution in pig muscle and the humoral immune response of pigs infected with moderate doses of this parasite; therefore, this study investigated the infectivity of a Polish strain of *T. britovi* for pigs, the antibody response of this host to various doses of *T. britovi,* and the efficiency of three different commercial ELISA kits and an immunoblot assay at detecting anti-*T. britovi* IgG. No significant differences in terms of the infection level of particular muscles or of whole carcasses between pigs infected with 3000 and those infected with 5000 ML of *T. britovi* were observed. The highest intensity of *T. britovi* infection was reported in the diaphragm pillars. The larvae of *T. britovi* showed homogeneous distribution with respect to the muscle side. Statistically, specific IgG antibodies against excretory–secretory (ES) antigens of *Trichinella* ML were first detected by all ELISA protocols on day 36 post infection; however, individual pig results showed some differences between the three tests applied. A significant increase in the level of anti-*T. britovi* IgG was observed between days 36 and 41 post infection, and from day 45 until day 62 after *T. britovi* infection, production of these antibodies reached its plateau phase. No positive correlation was found between the anti-*T. britovi* IgG level and the larvae density in 15 different muscles. Sera of *T. britovi*-infected pigs showed reactivity with *T. britovi* ML ES antigens of 62, 55, and 52 kDa. The results provide novel information on spatial larvae distribution in muscles and the humoral immune response of pigs exposed to two different doses of a Polish strain of *T. britovi*, extend knowledge on serological diagnostic tools which may be introduced in veterinary practice for the detection of *T. britovi* infections in pig production, and offer practical solutions for meat hygiene inspectors in the field at sampling sites when examining pig carcasses for *Trichinella*.

## 1. Introduction

Trichinellosis is an emerging and re-emerging worldwide foodborne zoonotic disease caused by the consumption of raw or undercooked meat infected with parasitic nematodes of the genus *Trichinella*. Globally, cases and outbreaks of human trichinellosis are primarily associated with the consumption of infected meat and/or meat products derived from domestic pigs and wild boars [1].

Among the 10 named and known species of *Trichinella* (*Trichinella spiralis*, *Trichinella nativa*, *Trichinella britovi*, *Trichinella pseudospiralis*, *Trichinella murrelli*, *Trichinella nelsoni*, *Trichinella papuae*, *Trichinella zimbabwensis*, *Trichinella patagoniensis*, and *Trichinella chanchalensis*) and the three characterized genotypes (T6, T8, and T9) [2,3,4], *T. spiralis*, *T. britovi*, and *T. pseudospiralis* have been confirmed as the causative agents of natural *Trichinella* infections in European breeding pigs. Infections induced by *T. pseudospiralis* were only confined to single pigs in Slovakia, Croatia, and Bosnia and Herzegovina [5,6,7]. In contrast, natural infections of domestic swine have been proven to be caused by both *T. spiralis* and *T. britovi* in many countries in Europe; however, the frequency of their occurrence differs significantly. In general, *T. spiralis* is more prevalent than *T. britovi* in the European domestic pig population [8]. According to data obtained from 22 European countries and compiled by the International Trichinella Reference Centre, 18% of all *Trichinella* isolates from infected pigs were identified as *T. britovi*, while the corresponding figure for *T. spiralis* was 82% [8]. These proportions are different, however, in those countries that have eliminated the domestic transmission cycle that maintains *T. spiralis* circulation. Long-term studies performed in Poland in 2009–2016 showed that *T. britovi* was the causative agent of 1% of the total *Trichinella* infections of pigs [9]. The same trend in *Trichinella* species distribution was shown in wild boars; however, the ratio of *T. spiralis* prevalence to that of *T. britovi* is decidedly lower in boars, and in Poland is estimated to be 3:1 [9,10].

*Trichinella britovi* (genotype T3) is characterized by a wide geographic range of occurrences and its main natural hosts are wild carnivores living in temperate regions of Europe, Western Asia, and Northern and Western Africa [11]. The transmission cycle of *T. britovi* is therefore sylvatic; however, it has also been proven that this species can be transferred from wildlife to domestic pigs [11]. Such transmission is particularly possible in areas where pigs are kept in free-range, backyard, or organic breeding systems that allow them to contact other wild animals unhindered. Several mechanisms that lead to the infection of pigs with *Trichinella* nematodes can be indicated. One is the feeding of animals with fragments or whole carcasses of carnivores infected with *Trichinella* larvae [12] that may proceed from unsanctioned hunting and/or breeding practices. Taking into account the relatively high prevalence of *Trichinella* among foxes in Poland (4%), with *T. britovi* being the dominant species [9], and the typical location where *Trichinella* infections in pigs are found being small farms with poor biosecurity systems and herd sizes of no more than 35 animals [13], it should be assumed that *T. britovi* may start to play an increasingly important role in the natural infections of pigs. In fact, the results of some studies proved that *T. britovi* shows low infectivity for domestic pigs and rats [14,15,16,17]. This allowed a paradigm to be formulated in which the spread of this parasite on the farm in the pig–pig or rat–pig chain model is limited and, therefore, *T. britovi* foci in pigs are rather short-lived and confined to one generation of breeding swine. However, our previous study performed in a *T. britovi*-infected swine model [18] showed that moderate doses of *T. britovi* were able to induce swine infection, which met the conditions of creating a risk to public health (mean infection intensity > one larva in 1 g of muscle tissue) and having potential for spreading to other individuals in the herd. Because of *T. britovi*’s widespread circulation among various species of wild omni- and carnivorous animals in Europe, the possibility of its transmission from wildlife to the pig production chain, and serious documented outbreaks in humans [19,20,21], this species should be prioritized in the *Trichinella* genus in the One Health approach to zoonotic foodborne parasites.

In order to better understand the effect of various doses of a Polish strain of *T. britovi* on host–parasite interactions in pigs as well as to assess different serological tests for the detection of *T. britovi* infections in pigs, the objectives of this study were to: (i) evaluate and compare the intensity and distribution of *T. britovi* ML between 15 different muscles and different sides of the same muscles of a native Polish breed of pigs experimentally inoculated with two different doses of *T. britovi*, (ii) evaluate different ELISA kits commercialized in Europe based on excretory–secretory (ES) antigens of *Trichinella* ML for the detection of *T. britovi* infection in pigs, (iii) assess the kinetics of the anti-*T. britovi* IgG response (production increases and/or decreases) in short time intervals during a 62-day period of infection, (iv) identify the pattern of *T. britovi* ML ES protein reaction with pig sera using Western blot, and (v) compare a Western blot based on ES antigens from *T. britovi* with ELISA tests in terms of detection of seroconversion in *T. britovi*-infected swine.

## 2. Results

### 2.1. Intensity of T. britovi ML Infection and Distribution of the Larvae in Muscles of Pigs Experimentally Infected with Different Doses of T. britovi

The distribution of *T. britovi* ML in particular muscles and muscle sides of pigs experimentally infected with 3000 and 5000 ML of *T. britovi* is shown in Table 1 and Table 2. Muscle larvae of *T. britovi* were isolated from all experimentally infected pigs and the presence of the larvae was confirmed in all muscles analyzed. The average larval load in the entire carcasses of pigs infected with 3000 ML was 13.84 lpg, while in pigs infected with a dose of 5000 ML it was 14.34 lpg.

Muscle larvae of *T. britovi* showed a homogeneous distribution with respect to the muscle side, since no statistically significant differences were found between the left and right sides of the 13 different laterally paired muscles in both experimental groups of pigs (Student’s *t*-test, *p* ˃ 0.05). The highest intensity of *T. britovi* ML infection was reported in the diaphragm pillars, and, with the exception of the tongue, it significantly differed from the intensity of infection of the remaining 13 muscles in the group of pigs infected with 3000 ML (one-way ANOVA followed by Tukey’s post hoc test, *p* ˂ 0.0001) or 5000 ML of *T. britovi* (Welch’s ANOVA followed by Games–Howell’s post hoc test, *p* ˂ 0.0001).

There were no significant differences in the infection level of particular muscles or entire carcasses between pigs infected with 3000 and those infected with 5000 ML of *T. britovi* when a Mann–Whitney *U*-test was applied (*p* ˃ 0.05) (Table 3).

### 2.2. Detection of Anti-T. britovi IgG in Pigs Using Three Commercial ELISA Kits

The specific IgG antibody response against the ES antigens of *Trichinella* ML measured using different ELISA commercial kits in the group of pigs experimentally infected with 5000 ML of *T. britovi* is shown in Table 4, and a serological evaluation of individual *T. britovi*-infected pigs is presented in Table 5. Statistically, all three ELISA tests first detected IgG antibodies against the *T. britovi* ML ES antigens (PP ≥ 15% for the PrioCHECK^®^ *Trichinella* Ab ELISA (ThermoFisher Scientific, Schlieren, Switzerland); S/P% ≥ 60% for the ID Screen^®^ *Trichinella* Indirect Multi-species ELISA (ID Vet, Grabels, France); and S/P ≥ 0.3 for the Pigtype^®^ *Trichinella* Ab ELISA (Qiagen, Leipzig, Germany)) on day 36 post infection (pi) (Table 4). Among eight infected pigs, four (pigs no. 1, 5, 6, and 8) were first found to be positive by all three tests on the same day after infection (36 or 41 dpi) (Table 5). In the case of another two pigs (pigs no. 3 and 4), the PrioCHECK^®^ *Trichinella* Ab and ID Screen^®^ *Trichinella* Indirect Multi-species assays gave a positive result on the same day after inoculation (45 and 29 dpi, respectively), whereas the Pigtype^®^ *Trichinella* Ab ELISA classified these pigs as positive one blood draw earlier (41 and 27 dpi, respectively) (Table 5). Consistency between the results given by the PrioCHECK^®^ *Trichinella* Ab test and the Pigtype^®^ *Trichinella* Ab test was observed in pigs no. 2 and 7, which were found to be positive on days 36 and 41 pi, respectively, while the ID Screen^®^ *Trichinella* Indirect Multi-species ELISA classified these pigs as positive one blood draw later (41 and 45 dpi, respectively) (Table 5). In addition, two pig serum samples (from pigs no. 2 and 7 collected on days 36 and 41 pi, respectively) which gave doubtful results in the ID Screen^®^ *Trichinella* Indirect Multi-species ELISA with relatively high S/P% values (51.770 and 56.163%) were classified as positive by the other two tests on those days. Moreover, serum samples obtained from five pigs (pigs no. 1, 2, 3, 4, and 5) on days 27, 29, 36, and 41 pi were classified either by the PrioCHECK^®^ *Trichinella* Ab or Pigtype^®^ *Trichinella* Ab assay as negative, but their PP or S/P value reached a level close to the cut-off (i.e., PP = 13.057%, 14.248%, and 14.810%, respectively for pigs no. 3, 4, and 5 analyzed by the PrioCHECK^®^ *Trichinella* Ab assay and S/P = 0.287, 0.288, and 0.288 for pigs no. 1, 2, and 3 analyzed by the Pigtype^®^ *Trichinella* Ab kit). As expected, all these pigs, including those that generated doubtful results in the ID Screen^®^ *Trichinella* Indirect Multi-species ELISA, were detected as positive at the next blood collection time point (i.e., 2, 4, 5, or 7 days later) by all the ELISA kits applied.

### 2.3. Variation in the Level of Anti-T. britovi IgG in the Serum of Pigs Infected with T. britovi

In the group of pigs experimentally infected with 5000 ML of *T. britovi*, a statistically significant increase in the level of anti-*Trichinella* IgG was observed between days 36 and 41 post infection when all three assays were performed. The significance is demonstrated for the ID Screen^®^ *Trichinella* Indirect Multi-species ELISA results by *p* ˂ 0.0001 in Mauchly’s sphericity test, *p* = 0.007 in Greenhouse–Geisser’s correction, *p* = 0.005 in Huynh–Feldt’s correction, *p* = 0.01 by lower bound estimation, and *p* = 0.002 in the Bonferroni post hoc test. For the Pigtype^®^ *Trichinella* Ab ELISA results, the corresponding values are *p* ˂ 0.001 in Mauchly’s sphericity test, *p* ˂ 0.0001 in Greenhouse–Geisser’s correction, *p* ˂ 0.0001 in Huynh–Feldt’s correction, *p* = 0.004 by lower bound estimation, and *p* ˂ 0.0001 in the Bonferroni post hoc test. The PrioCHECK^®^ *Trichinella* Ab ELISA results yielded *p* ˂ 0.0001 in Mauchly’s sphericity test, *p* = 0.0006 in Greenhouse–Geisser’s correction, *p* = 0.0002 in Huynh–Feldt’s correction, *p* = 0.005 by lower bound estimation, and *p* = 0.006 in the Bonferroni post hoc test (Table 4). In general, from day 45 until the end of the experiment, i.e., day 62 after *T. britovi* infection, the production of these antibodies reached a plateau phase and persisted at a constant high level without any significant differences as measured by all three ELISA tests (Table 4).

### 2.4. Correlation between Number of Recovered Larvae (lpg) in Particular Muscles of Experimentally Infected Pigs and Anti-T. britovi IgG Level

No positive correlation was found between larvae density in 15 different muscles and OD level measured by three different ELISA kits on days 41, 51, and 62 post infection, except for one partial example at 41 dpi when such a correlation was shown between the intensity of *T. britovi* ML infection in forelimb extensors and the OD value generated by the Pigtype^®^ *Trichinella* Ab assay (Table 6).

### 2.5. Correlations between ELISA Kits

Table 7 summarizes the correlations between ELISA index values (PP, S/P%, and S/P) generated by three different ELISA kits in individual pigs experimentally infected with *T. britovi* as well as the entire experimental model. In all eight pigs infected with 5000 ML of *T. britovi*, a significant (*p* ˂ 0.05) strong or very strong positive correlation with *R* correlation coefficients ranging from 0.725 to 0.895 and from 0.907 to 0.963, respectively, was found between the ELISA index values generated by the PrioCHECK^®^ *Trichinella* Ab ELISA (PP) and the ID Screen^®^ *Trichinella* Indirect Multi-species ELISA (S/P%); PrioCHECK^®^ *Trichinella* Ab ELISA (PP) and Pigtype^®^ *Trichinella* Ab ELISA (S/P); and ID Screen^®^ *Trichinella* Indirect Multi-species ELISA (S/P%) and Pigtype^®^ *Trichinella* Ab ELISA (SP). In addition, a high positive correlation with *R* correlation coefficient ranging from 0.903 to 0.912 was found between all three tests for the entire experimental model (Table 7).

### 2.6. Recognition of the T. britovi ML ES Antigens by Anti-T. britovi IgG in Western Blot

**Figure 1:**
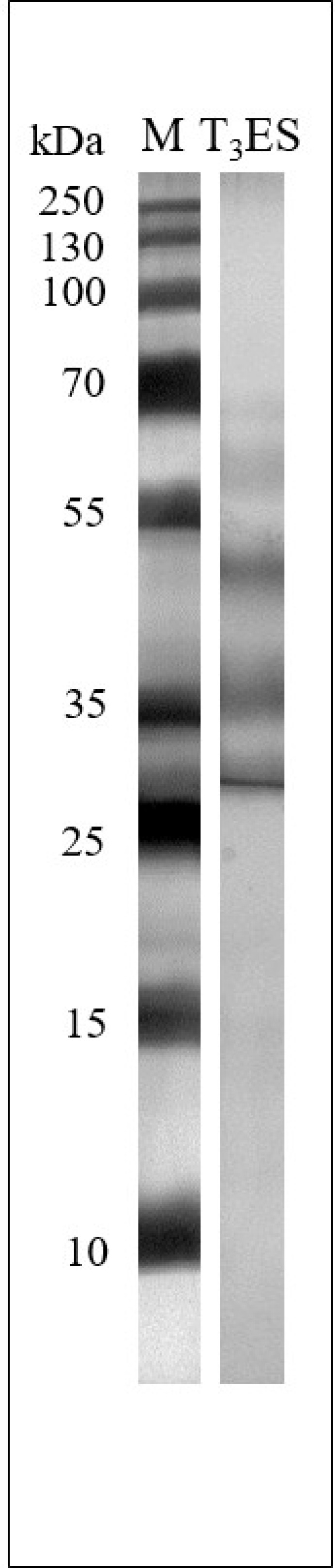
SDS-PAGE analysis of muscle larvae (ML) excretory–secretory proteins of *T. britovi* (T3 ES). M—molecular weight marker in kDa.

**Figure 2:**
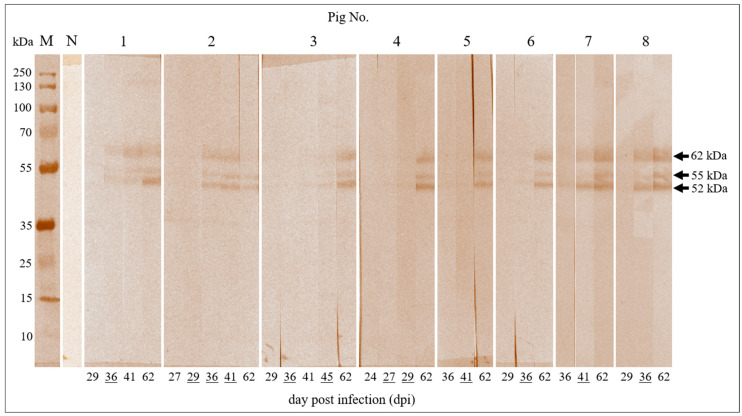
The immunoblot analysis of *T. britovi* ML ES proteins incubated with *T. britovi*-infected pig sera samples and the negative control sample. Each pig was tested separately. The following serum samples were used in immunoblot analysis: (1) pig sera from the day when the results generated by commercial ELISAs indicated seroconversion, (2) pig sera from one or two sampling time points earlier than the day of seroconversion, and (3) pig serum samples from day 62 post infection. M—molecular weight marker in kDa.; N—negative control (serum samples collected from pigs 4 days prior to experimental infection with *T. britovi*).

The electrophoretic pattern of the *T. britovi* ML ES antigen is shown in Figure 1, and the Western blot patterns of reactivity of the ES antigens with sera collected from individual pigs infected with 5000 ML of *T. britovi* at various time points after infection are presented in Figure 2. *T. britovi* ML ES antigens showed bands of molecular weights in the range of 20 to 95 kDa with the most intense signal between 30 and 48 kDa (Figure 1). Anti-*T. britovi* IgG antibodies in individual pig serum samples were first detected by Western blot on day 29 (in pig no. 4), 36 (in pigs no. 1, 2, 6, and 8), or 41 (pigs no. 3, 5, and 7) post infection. Serum samples from infected pigs recognized *T. britovi* ML ES antigens of molecular weights of 62, 55, and 52 kDa. The immunoblot indicated that all the protein bands observed on the day of seroconversion were also observable on successive days until the end of the experiment. Representative patterns also showed the differences in signal intensity and relative migration value of *T. britovi* ML ES reactive proteins between serum samples from individual pigs on particular days after infection. In general, all recognized bands in all experimentally infected pigs were more intense in the late phase of infection (62 dpi) than on the day of seroconversion. Sera collected from pigs before experimental infection did not recognize *Trichinella* protein bands.

## 3. Discussion

*Trichinella britovi* is widely distributed among wildlife and several studies have confirmed that this species predominates in red foxes, raccoon dogs, and wolves in Poland [9,22,23,24]. The parasite has also been found in martens and badgers living in the northeast regions of this country [25], which only proves its circulation in a wide range of hosts. Moreover, according to the Polish General Inspectorate, *Trichinella* is regularly detected in wild boars [26] and, as recent data have shown, almost 20% of all *Trichinella*-infested Polish wild boars were found to be infected with the T3 genotype [9,10]. Therefore, it should be stated that such high infection pressure exerted by environmental *T. britovi* poses a significant risk to pigs, particularly those in small backyard/outdoor breeding systems that are typically operated to poor zoohygienic standards. If the misconduct of hunters, who are often also the pig breeders, and the feeding of pigs with the offal and remains of hunted animals are included, the risk of *T. britovi* pig infection rises higher. Therefore, to better understand the infectivity of *T. britovi* for native Polish pig breeds and the humoral immune response accompanying the invasion, as well as to determine the usefulness of various commercial ELISA kits in the diagnostics of swine infection induced by various doses of *T. britovi*, we decided to extend our previous research [18] and provide deeper insight into *T. britovi* infection mechanisms in pigs. To achieve these goals, we used native Polish pig breeds and a Polish strain of *T. britovi* that had been isolated from a naturally infected wild boar and passaged at least once through a domestic pig before this experiment. Our results showed that in the group of pigs infected with 3000 and in the group infected with 5000 ML of *T. britovi*, the diaphragm pillars were the most heavily parasitized muscle and, with the exception of the tongue, the infection level in the diaphragm pillars was significantly higher than the level in the other 13 muscles analyzed. This shows that the mechanism of *T. britovi* larvae deposition in swine muscles is rather similar to that of *T. spiralis*, and the diaphragm pillars should be considered the predilection site during official pig carcass examination for *Trichinella*. There are only three comprehensive studies on the distribution of *T. britovi* ML in pig muscles for comparison purposes. Nöckler et al. [16] showed that in pigs experimentally infected with 200, 1000, or 20,000 ML of *T. britovi*, the highest larvae burden was observed in the diaphragm. In contrast, in the studies performed by Pozio et al. [17], the tongue was the most highly parasitized muscle in swine experimentally infected with 10,000 ML of *T. britovi*. Other studies conducted by Kapel et al. [14] showed that, in pigs infected with 10,000 ML of *T. britovi*, the tongue tip presented the highest larvae density out of 18 muscles analyzed. Recent changes to the Regulation (EU) 1375/2015 [27] introduced the ISO 18743:2015 standard, in which the reference detection method of *Trichinella* larvae in meat as well as the sampling procedure are described in detail [28]. According to this document, in the case of official examination of domestic pigs for *Trichinella*, muscle samples should be taken from the diaphragm pillars or masseters. Our study results, however, showed that in pigs infected with 3000 ML and 5000 ML of *T. britovi*, the mean larval density in the masseters amounted to, respectively, 51.93% and 36.11% of that observed in the diaphragm pillars. In the studies performed by Nöckler et al. [16], this percentage ranged from 18.50 to 60.74 and in the research conducted by Pozio et al. [17] it was 70.59. It is difficult to speculate whether the same proportions would be observed with the intensity of infection of *T. britovi* in the diaphragm pillars at the level of the sensitivity of the artificial digestion method (i.e., one larva per 1 g of diaphragm pillars); however, Prost and Nowakowski [29] have shown that in pigs in which the intensity of infection in the diaphragm pillars ranged from 0.91 to 1.18 lpg of *T. spiralis*, the mean larval density in masseter muscles was only 21.2% of that observed in the pillars. This may suggest that the masseter muscles should not always be considered as equivalent to diaphragm pillars, and that the mass of the samples taken from masseters for official pig carcass examination should be proportionally increased, particularly when the maximum of 100 carcasses are tested in one batch and each animal is represented by a sample mass of 1 g. Furthermore, in all 13 laterally paired pig muscles analyzed, *T. britovi* showed a homogeneous distribution in terms of both anatomical location and the muscle side. Our study also showed that despite the use of two different doses of *T. britovi*, no exponential increase of larvae burden was observed in entire infected pig carcasses. In general, the *Trichinella* muscle larvae density correlates with the ingested dose of the parasites. However, such interdependencies are most apparent when the magnitude of the infective doses vary greatly and fall in different categories such as low (100–300 ML), moderate (2000–5000 ML), or high (10,000–20,000 ML) in terms of total number of larvae used for the infection. In other words, the use of doses of various categories induces significant differences in the muscle larval load, but various doses from one category do not always produce such results. The similar infection level in pigs infected with different doses of *T. britovi* detected in this study may be explained by a stronger local immune response elicited by the dose of 5000 ML and, as a consequence, more rapid expulsion of worms from the swine intestines. In the global literature, there are few data available that describe how different doses of *T. britovi* affect the larvae density in pig muscles. Nöckler et al. [16], for instance, showed that despite the use of two different infective doses, i.e., 200 and 1000 ML of *T. britovi*, the larvae burden in infected pig carcasses was rather similar and amounted to 1.82 and 0.98 lpg, respectively. In this context, important information was also provided by Franssen et al. [30], who showed that in rats experimentally infected with *T. spiralis*, increasing doses resulted in increasing numbers of recovered larvae only in a non-linear manner and doses above 10,000 ML even resulted in decreasing larvae density.

Another aim of the current work was to assess the antibody response in pigs infected with 5000 ML of *T. britovi* by using various commercial ELISA tests. Statistically, specific IgG antibodies against the ES antigens of *Trichinella* ML were first detected by all ELISA protocols on day 36 pi; however, individual pig results differed between the three tests applied. It is well known that the sensitivity of the serological tests depends on the time that has elapsed from the infection. The sensitivity of the PrioCHECK^®^ *Trichinella* Ab and Pigtype^®^ *Trichinella* Ab ELISAs was 62.5% (95% CI: 24.49–91.48) on day 36 pi, while for ID Screen^®^ *Trichinella* Indirect Multi-species it was 50% (95% CI: 15.7–84.30). The highest sensitivity on day 41 pi was shown by the Pigtype^®^ *Trichinella* Ab ELISA (100%, 95% CI: 63.06–100), while the PrioCHECK^®^ *Trichinella* Ab and ID Screen^®^ *Trichinella* Indirect Multi-species assays showed 87.5 (95% CI: 47.35–99.68) and 75% (95% CI: 34.91–96.81), respectively. The variations in individual pig classification between tests as positive, doubtful, or negative may be associated with the differences in the ELISA reagents, i.e., the methods of the ES antigen production, the determined cut-off point, or the type of secondary antibody used. In this regard, our previous study showed that an in-house ELISA based on *T. britovi* ML ES antigens was able to detect anti-*Trichinella* IgG in a group of pigs experimentally infected with 3000 ML of *T. britovi* one blood sampling earlier (6 days) than both the commercial PrioCHECK^®^ *Trichinella* Ab ELISA and an in-house *T. spiralis* ML ES-antigen ELISA. In addition, from a practical point of view, special attention should be paid to doubtful results, or, if the ELISA is construed to yield only positive (specific anti-*Trichinella* IgG detected) or negative (specific anti-*Trichinella* IgG not detected) results, consideration should be given to the serum OD values that are close to the cut-off and, in particular, those that reach 90% of the cut-off value and above. In such a scenario, the pigs should be re-tested and this examination may be performed over a very short time interval, e.g., 2–5 days. The wisdom of treating certain results with caution is also supported by our trial study in which serum samples from 100 healthy fatteners weighing 80–100 kg and aged 6–9 months without *Trichinella* larvae in 1 g muscle generated ELISA index values of 15, 4.43, and 13.67% of the cut-off value in the PrioCHECK^®^ *Trichinella* Ab, ID Screen^®^ *Trichinella* Indirect Multi-species, and Pigtype^®^ *Trichinella* Ab ELISAs, respectively (data not shown). It should also be noted that the differences in the detection of *T. britovi* infection between the ELISA tests in individual pigs concerned no more than a single blood collection time point. As serum samples were drawn at 2–6-day intervals, it can be assumed that the ELISA kit that generated a negative result, while the other ELISA kit produced a positive one, would have yielded a positive result even on the next day of blood collection. Overall, the correlations between ELISA kits were high for both individual infected pigs and the entire experimental model. Our research results also fill a gap in the literature, as, in the few existing studies, most authors used high doses of *T. britovi* to examine the immune response of experimentally infected pigs. In pigs infected with high doses of *T. britovi* (10,000–20,000) of various larval densities (2.23–123.1), seroconversion was observed between day 21 and 42 after inoculation [14,16,17,31]. At the low end of the dose scale, pigs infected with 200 or 1000 ML (three pigs per group) of *T. britovi* seroconverted at 60 dpi [16].

Finally, our study showed that a statistically significant increase in the level of anti-*T. britovi* IgG was observed between days 36 and 41 post infection and that from day 45 until the end of the experiment, i.e., day 62 after *T. britovi* infection, production of these antibodies reached its plateau phase and persisted at a constant high level without any significant differences as measured by all three ELISA tests. Similar kinetics in antibody production were observed in our previous experiment, when pigs were infected with 3000 ML of *T. britovi* [18]. These results indicate that the anti-*T. britovi* IgG production kinetics are not dose-dependent in pigs infected with different doses in the range of 3000–5000 ML of *T. britovi*. Moreover, when these results are compared with our previous ones [18], there were no statistically significant differences in the level of anti-*T. britovi* IgG antibodies between the groups of pigs infected with a dose of 3000 and 5000 larvae on days 36 and 45 post infection. The day 36 PrioCHECK^®^ *Trichinella* OD values were 0.661 ± 0.190 and 1.034 ± 0.801 for pigs experimentally infected with 3000 and 5000 ML of *T. britovi*, respectively, and a Mann–Whitney *U* test gave a *p* value of 0.747. The day 45 values after the same ELISA were 1.404 ± 0.278 and 2.038 ± 0.653 for pigs experimentally infected with 3000 and 5000 ML of *T. britovi*, respectively, and a Mann–Whitney *U* test gave a *p* value of 0.081. Nevertheless, anti-*T. britovi* IgG antibody level differences were found on day 62 pi, when the ELISA mentioned previously returned OD values of 1.627 ± 0.358 and 2.434 ± 0.481 for pigs experimentally infected with 3000 and 5000 ML of *T. britovi*, respectively, and a Mann–Whitney *U* test produced a *p* value of 0.01. This phenomenon is difficult to explain, because the infection levels in the two groups of pigs did not differ significantly. In addition, the differences in absolute anti-*T. britovi* IgG level between the group of pigs infected with 3000 and the group infected with 5000 ML of *T. britovi* were found only in the late parenteral phase (62 dpi) of the infection, which, in fact, also excludes any dose effect. It can therefore be assumed that there are endogenous factors which regulate the amount of protein products excreted or secreted by the parasite into the host’s bloodstream in an unknown manner and that, so regulated, these protein products stimulate the host’s immune system with varying intensity. This is also supported by the lack of correlation between the larvae density in different muscles of the infected swine and the anti-*T. britovi* level on particular days after infection.

In general, the Western blot showed high compliance with the PrioCHECK^®^ *Trichinella* Ab and Pigtype^®^ *Trichinella* Ab ELISA results. In the cases of three pigs, the Western blot detected anti-*T. britovi* IgG one blood collection earlier than the ID Screen^®^ *Trichinella* Indirect Multi-species ELISA kit. However, it should also be highlighted that in the case of these serum samples, the ID Screen^®^ *Trichinella* Indirect Multi-species assay generated doubtful results with relatively high ELISA index values. Moreover, our immunoblot results confirmed the differences in anti-*T. britovi* IgG level during the course of infection, which were observed in all three ELISA tests: the signal intensity of ES protein bands reacting with anti-*T. britovi* IgG varied and was significantly higher in the late phase of infection (62 dpi) than on the day of seroconversion. The swine sera reacted with *T. britovi* ML ES antigens with molecular weights of 62, 55, and 52 kDa. In fact, our results are partially in accordance with the comprehensive studies performed by Gómez-Morales et al. [32], who showed that *Trichinella*-infected pig sera recognized ES proteins with a three-band pattern of 64–72, 59–63, and 48–55 kDa. It should be noted, however, that all global studies on the use of Western blot for the detection of *Trichinella* infection in pigs were performed using *T. spiralis* ML ES antigens as well as serum samples collected from pigs infected with *T. spiralis* [32,33]; the results of this research have been discussed elsewhere [34]. It was also proved that all *Trichinella* species share some ES proteins and, as a consequence, an ES antigen obtained from one *Trichinella* species is able to detect infections induced by other *Trichinella* species and genotypes [31]; however, recently, Grzelak et al. [35] showed that sera from *Trichinella*-infected persons displayed some differences in terms of their specific band patterns when *T. spiralis* and *T. britovi* ML ES antigens were used. It was also found by using one-dimensional electrophoresis that the native *T. spiralis* ES antigen shows more protein bands than the *T. britovi* ML ES antigen [35]. However, on the basis of our results and studies conducted by Gómez Morales et al. [32], it should be stated that the use of *T. britovi* ML ES antigen and serum samples of pigs infected with *T. britovi* did not significantly affect the Western blot band pattern compared with the pattern generated by the serum of pigs infected with *T. spiralis* tested by using *T. spiralis* ES-WB. Our studies have also shown that the Western blot pattern of individual infected pigs was stable; ES proteins that reacted with anti-*T. britovi* IgG on the day of seroconversion were also visualized after 20–30 days (i.e., 62 days after infection). Likewise, there were no additional protein bands reacting with anti-*T. britovi* IgG in the late phase of infection (day 62 pi) which had not been observed on the day of seroconversion. This may indicate that the invasion phase in particular individuals infected with *T. britovi* has no effect on the protein band profile assessed by using one-dimensional electrophoresis and a Western blot based on ES antigens from the muscle larvae of *T. britovi*. Therefore, for the purpose of wide-scale method validation studies, in which a Western-blot pattern is established, it is sufficient to take the sample from particular individuals at one time point after infection, provided that seroconversion is known to have occurred.

## 4. Materials and Methods

### 4.1. Parasites, Animals, and Study Design

*Trichinella britovi* ML were isolated in our laboratory during routine inspections of meat from a naturally infected wild boar and then identified at the species level by multiplex polymerase chain reaction (multiplex PCR) according to the method described by Zarlenga et al. [36]. Muscle larvae (ML) displaying motility were counted and then suspended in 30% gelatin blocks. Fourteen healthy young Puławska/Polish Large White crossbreed pigs aged 10 weeks and of 20 kg average body weight were purchased from the Czesławice experimental farm belonging to the University of Life Sciences in Lublin. The animals were randomly divided into two experimental groups (A1, n = 8 and A2, n = 6) and subsequently infected by administering a single dose *per os* of 5000 *T. britovi* ML/pig to Group A1 or 3000 *T. britovi* ML/pig to Group A2. Blood samples from each pig in Group A1 were drawn from the right external jugular vein at 4 days prior to *T. britovi* experimental infection (−4) and at 3, 6, 9, 13, 15, 17, 20, 24, 27, 29, 36, 41, 45, 51, 55, 59, and 62 days post infection (dpi). Blood samples from pigs in Group A2 were collected in the same way at 5 days prior to the infection and at 6, 13, 20, 30, 36, 45, and 62 dpi, and then they were designated for other serological studies as a part of broader research project conducted by our laboratory. During the experiment, pigs were housed in separate units with free access to water. The pigs were fed twice daily. The feed was prepared at an experimental farm of the University of Life Sciences in Lublin and was based on wheat (50%) and barley (35%) supplemented (15%) with protein concentrate, vitamins, and minerals.

### 4.2. Larval Recovery and Counting

Pigs were sacrificed 62 days after experimental inoculation. The muscle larval burden was determined by the digestion procedure laid down in ISO 18743:2015 standard [28]. The samples intended for the digestion assay were collected from the diaphragm pillars (*crura diaphragmatica*), tongue (*lingua*), and the following muscles or muscle groups from the left and right sides of each carcass: the masseter (*M. masseter*), lateral and medial pterygoid (*M. pterygoideus lateralis et medialis*), intercostals (*Mm. intercostales*), abdomen (*Mm. abdominis*), back (*M. longissimus thoracis*), neck (*Mm. colli*), sublumbar (*M. psoas major et minor*), triceps brachii (*M. triceps brachii*), femoral biceps (*M. biceps femoris*), forelimb extensors (*Mm. extensores antebrachii*), forelimb flexors (*Mm. flexores antebrachii*), crus extensors (*Mm. extensores cruris*), and crus flexors (*Mm. flexores cruris*). The diaphragm pillars, tongue, and masseters were digested entirely. The remaining muscles of total weight under 50 g were digested entirely, whereas those over 50 g were cropped to form 50 g specimens and then digested. The number of muscle larvae per gram of muscle tissue (lpg) was presented as an average calculated separately for Group A1 and Group A2 and each muscle examined.

### 4.3. Serological Analyses

The serological analyses described below were performed for the group of pigs infected with 5000 ML of *T. britovi* (Group A1). The serological results from pigs infected with 3000 ML of *T. britovi* (Group A2) generated by using an in-house ELISA based on *T. spiralis* and *T. britovi* ML ES antigens and a PrioCHECK^®^ *Trichinella* Ab ELISA were described in detail in our previous paper as a part of broader research cycle [18].

#### 4.3.1. Commercial ELISA Tests for Anti-*T. britovi* IgG Detection

Serum levels of specific IgG antibodies against *Trichinella* ML ES antigens were determined using three different commercial diagnostic ES ELISA kits that are available on the European Union market: the PrioCHECK^®^ *Trichinella* Ab (ThermoFisher Scientific, Schlieren, Switzerland); the ID Screen^®^ *Trichinella* Indirect Multi-species (ID Vet, Grabels, France); and the Pigtype^®^ *Trichinella* Ab (Qiagen, Leipzig, Germany). All tests are based on *Trichinella* ES antigens. The PrioCHECK^®^ *Trichinella* Ab ELISA is suitable only for the examination of serum or meat juice samples from pigs, and peroxidase-labeled anti-pig antibody serves as a secondary antibody in this kit. The ID Screen^®^ *Trichinella* Indirect Multi-species and Pigtype^®^ *Trichinella* Ab ELISAs allow testing swine and other species of animals, and anti-*Trichinella* antibodies are detected by a multi-species horseradish peroxidase (HRP) conjugate. The results of the PrioCHECK^®^ *Trichinella* Ab and Pigtype^®^ *Trichinella* Ab tests are interpreted as positive or negative only. The design of the ID Screen^®^ *Trichinella* Indirect Multi-species ELISA allows doubtful results to be obtained as well. All analyses were performed according to the manufacturers’ instructions. The sera from uninfected and *T. britovi*-infected pigs and the controls (from the ELISA kits) were tested in duplicate; the final result was the mean of two measurements of optical density (OD) for each serum analyzed. All ELISA kits met the validation acceptance criteria that were set by the producer. Interpretation of the results for each particular ELISA kit was performed as follows:

PrioCHECK^®^ *Trichinella* Ab: the results were graded by calculating the percentage of positivity (PP) value according to the following formula:

$$\mathrm{PP}=\frac{\mathrm{OD}\;\left(\mathrm{Sample}\right)}{\mathrm{OD}\;\left(\mathrm{Positive}\;\mathrm{control}\right)\;}\times 100\%$$

Serum samples with a PP value equal to or over 15% (PP ≥ 15%) were classified as positive (specific anti-*Trichinella* IgG detected), while those with PP values lower than 15% (PP ˂ 15%) were considered negative (specific anti-*Trichinella* IgG not detected).

Pigtype^®^ *Trichinella* Ab ELISA: the results were categorized by calculating the sample-to-positivity (S/P) value according to the following formula:

$$S/P\;=\frac{\mathrm{OD}\;\left(\mathrm{Sample}\right)-\mathrm{OD}\;\left(\mathrm{Negative}\;\mathrm{control}\right)}{\mathrm{OD}\;\left(\mathrm{Positive}\;\mathrm{control}\right)-\mathrm{OD}\;\left(\mathrm{Negative}\;\mathrm{control}\right)}$$

Serum samples with S/P value equal to or over 0.3 (S/P ≥ 0.3) were regarded as positive (specific anti-*Trichinella* IgG detected), while those with S/P values lower than 0.3 (S/P ˂ 0.3) were noted as negative (specific anti-*Trichinella* IgG not detected).

ID Screen^®^ *Trichinella* Indirect Multi-species ELISA: the results were judged by calculating the S/P% value according to the following formula:

$$S/P\%\;=\frac{\mathrm{OD}\;\left(\mathrm{Sample}\right)-\mathrm{OD}\;\left(\mathrm{Negative}\;\mathrm{control}\right)}{\mathrm{OD}\;\left(\mathrm{Positive}\;\mathrm{control}\right)-\mathrm{OD}\;\left(\mathrm{Negative}\;\mathrm{control}\right)}\times 100$$

Serum samples with S/P% value equal to or over 60% (S/P% ≥ 60%) were logged as positive (specific anti-*Trichinella* IgG detected), while those with S/P% values equal or lower than 50% (S/P% ≤ 50%) were taken to be negative (specific anti-*Trichinella* IgG not detected). Serum samples with S/P% values lower than 60% but greater than 50% (50% ˂ S/P% ˂ 60%) were interpreted as doubtful. When doubtful results were obtained, the serum samples were retested and the results yielded after the second test were accepted for further analysis.

#### 4.3.2. Production of the *T. britovi* ML ES Antigen and Western Blot

The *T. britovi* ML ES antigen was produced as described previously [35,37] and kept frozen at −70 °C. Briefly, *T. britovi* ML were washed with Roswell Park Memorial Institute medium (RPMI)-1640 and resuspended at 5000 ML/mL in RPMI-1640 supplemented with 20 mM N-2-hydroxyethylpiperazine-N-2-ethane sulfonic acid (HEPES), 200 mM L-glutamine, 100 mM Na-pyruvate, and 100 units each of penicillin and streptomycin. Larvae were then incubated in a T-75 culture flask in 5% CO_2_ at 37 °C for 18 h. Following incubation, the medium containing ML ES products was filtered through a 0.22 μm membrane and concentrated by lyophilization. The protein concentration was determined using a NanoDrop 2000 spectrophotometer (Thermo Fisher Scientific, Waltham, MA, USA). Concentrate in 100 µg amounts was loaded onto 12% acrylamide separating gels. Electrophoresis by SDS-PAGE was run using a Mini-PROTEAN Tetra Cell electrophoresis chamber (Bio-Rad Laboratories, Hercules, CA, USA) at 180 V for approximately 1 h. PageRuler Unstained Protein Ladder (Thermo Fisher Scientific, Waltham, MA, USA) was loaded onto each gel as a weight marker. Fragments of gel were stained with a PlusOne Silver Staining Kit (GE Healthcare, Waukesha, WI, USA) according to the manufacturer’s protocol. The unstained gels were also used for immunoblotting. The ES antigens of *T. britovi* that had been separated on gels were transferred to nitrocellulose membranes (BioRad) through a wet transfer system (BioRad) at 95 V for 1 h. Subsequently, the membranes were blocked with Pierce Protein-Free T20 (TBS) Blocking Buffer (Thermo Fisher Scientific) for 1 h at room temperature and then cut into strips and incubated overnight at 4 °C with a set of *T. britovi*-infected pig sera diluted 1:100. In order to examine the protein band pattern at different time of infection, the following serum samples were used in immunoblot analysis: pig sera from the day when the results generated by commercial ELISAs indicated seroconversion, pig sera obtained one or two sampling time points earlier than the day of seroconversion detected by the ELISA tests, and pig serum samples obtained on day 62 post infection. The secondary antibody, goat anti-pig IgG conjugated to HRP (Sigma-Aldrich, St. Louis, MO, USA), was diluted 1:20,000 in PBS with 5% skimmed milk, added to each strip, and incubated for 1 h at room temperature. The immunoreactive bands were visualized on membrane strips using Sigmafast 3,3′-Diaminobenzidine tablets (Sigma-Aldrich) according to the manufacturer’s protocol. The gel and the membranes were scanned with the ChemiDoc™ MP Imaging System (Bio-Rad). The immunoblots were performed using two replicate samples. Serum samples collected from pigs 4 days prior to *T. britovi* infection were used as negative controls.

### 4.4. Statistical Analysis

Data are presented as means and standard deviations. Medians and interquartile ranges are also displayed, if necessary.

The obtained data were investigated for normality with a Shapiro–Wilk test and for homogeneity of variances with Levene’s test. In order to compare the intensity of infection in the left and right sides of the individual muscles or muscle groups within each group of pigs, Student’s *t*-test (all assumptions met) or a Mann–Whitney *U*-test (for non-normally distributed data) were used. In order to compare the intensity of infection in the 15 different muscles within each group of pigs, one-way analysis of variance (ANOVA) with a post hoc Tukey’s test (all assumptions met) or one-way ANOVA with Welch’s correction followed by a Games–Howell post hoc test (for data with unequal variances) were applied. Comparison of the intensity of *T. britovi* ML infection in particular muscles and the entire carcasses between the group of pigs infected with 3000 and the group infected with 5000 ML of *T. britovi* was performed using a Mann–Whitney *U*-test (where numbers of animals in groups were unequal).

The differences in anti-*T. britovi* IgG level during the course of infection within each ELISA test performed in the group of pigs infected with 5000 ML of *T. britovi* were calculated using repeated measures analysis of variance (ANOVA) with a post hoc Bonferroni test. Previously, sphericity had been assessed using Mauchly’s test. If the assumption of sphericity was violated, the Greenhouse–Geisser correction (Greenhouse–Geisser ԑ ˂ 0.75) or the Huynh–Feldt correction (Greenhouse–Geisser ԑ ˃ 0.75) were introduced. Comparison of the anti-*T. britovi* level on particular days after infection between the group of pigs infected with 3000 and the group infected with 5000 ML of *T. britovi* was performed by using a Mann–Whitney *U*-test (where numbers of animals in groups were unequal).

Correlation between the intensity of *T. britovi* ML infection in the muscles of pigs infected with 5000 ML of *T. britovi* and the level of anti-*T. britovi* IgG on days 41, 51, and 61 post infection was calculated using Pearson’s correlation coefficient.

Finally, correlations between the ELISA index values (PP, SP%, and SP) generated by the PrioCHECK^®^ *Trichinella* Ab and ID Screen^®^ *Trichinella* Indirect Multi-species ELISAs, PrioCHECK^®^ *Trichinella* Ab and Pigtype^®^ *Trichinella* Ab ELISAs, and ID Screen^®^ *Trichinella* Indirect Multi-species and Pigtype^®^ *Trichinella* Ab ELISAs for each pig infected with 5000 ML of *T. britovi* and the entire experimental model were calculated using Spearman’s correlation coefficient.

For all these analyses, the level of significance was set at *p* < 0.05. All statistical calculations were performed with Statistica (StatSoft, Kraków, Poland) and SPSS (SPSS Inc., Chicago, IL, USA) software.

## 5. Conclusions

Our data indicate that an inoculation dose in the range of 3000–5000 ML of a Polish strain of *T. britovi* is sufficient to induce infection in pigs with an infection level that may pose a threat to public health and that enables this parasite to spread in swine herds. Our results also confirmed that the diaphragm pillars should be considered a predilection site for sampling during routine post-mortem examination of pigs for *Trichinella*. The results also showed that the muscle side sampled has no relevance for the intensity of *T. britovi* ML determined, and they imply that in cases of epidemiological study or limited sample availability it is sufficient to take a sample from one side of an individual muscle and/or muscle group to obtain reliable results. Within the range of the doses of 3000—5000 ML of *T*. *britovi* used for pig infection, there are no clear interdependencies between the dose and the larval density in the muscles and this suggests that higher doses are effective in inducing the immune system to reduce the number of newborn larvae or to remove the parasites from the intestines in a more efficient manner. Further, in pigs inoculated with doses of 3000—5000 ML of *T. britovi*, an anti-*T. britovi* specific IgG response appears on day 36 pi, reaches a plateau on day 45 pi, and remains stable until 2 months pi. It should also be concluded that in pigs infected with moderate doses of *T. britovi*, there are other factors driving the specific anti-*T. britovi* production than the dose and the density of the larvae in muscles, because there were no differences in the kinetics of antibody production over the 62-day period of infection nor in the absolute levels of antibodies on days 36 and 45 pi between the group of pigs exposed to the dose of 3000 and the group exposed to 5000 ML; neither was there any correlation between the lpg value and the absolute level of antibodies on individual days after inoculation. The detectability of *T. britovi* infection in individual pigs differed slightly between the commercial ELISA kits on days 36 and 41 pi, which may be due to differences in the production of the kits’ ES antigens, the method of determining the cut-off value, or the type of secondary antibody used. Finally, we conclude that when an ELISA yields doubtful results or the generated ELISA index values are close to the cut-off level (90% of cut-off or above), the pigs must be retested; this can be carried out after a short time interval such as 2–5 days. A Western blot test based on *T. britovi* ML ES antigens confirmed the ELISA results, and the sera of *T. britovi*-infected pigs showed reactivity with *T. britovi* ML ES antigens of 62, 55, and 52 kDa.

## Figures and Tables

**Table 1:** Distribution and intensity of *T. britovi* larvae infection (lpg) in muscles of pigs (n = 6) experimentally infected with 3000 muscle larvae of *T. britovi*.

Muscle/Muscle Group	Numbers of *Trichinella* Larvae/g Muscle (lpg)			
	Side		*p* ^1^	Left and Right (Combined)
	Left	Right		
	Mean ± SD (Median; IQR)	Mean ± SD (Median; IQR)		Mean ± SD (Median; IQR)
Diaphragm pillars (*Crura diaphragmatica*)	-	-	-	41.46 ± 20.28
Tongue (*Lingua*)	-	-	-	37.75 ± 19.58
Masseter (*M. masseter*)	21.32 ± 11.22	21.72 ± 13.44	0.956 ^†^	21.53 ± 12.31 *
Neck (*Mm. colli*)	12.06 ± 9.00	14.53 ± 10.21	0.667 ^†^	13.30 ± 9.36 *
Triceps brachii (*M. triceps brachii*)	9.71 ± 3.63	10.56 ± 6.48	0.787 ^†^	10.14 ± 4.93 *
Lateral and medial pterygoid (*M. pterygoideus lateralis et medialis*)	17.16 ± 14.15	15.34 ± 10.93	0.808 ^†^	16.29 ± 12.73 *
Sublumbar (*M. psoas major et minor*)	9.42 ± 5.04	11.09 ± 8.03	0.675 ^†^	10.25 ± 6.53 *
Abdomen (*Mm. abdominis*)	12.02 ± 9.03	12.26 ± 7.61	0.960 ^†^	12.14 ± 8.32 *
Forelimb extensors (*Mm. extensores antebrachii*)	8.48 ± 4.96	8.65 ± 6.90	0.963 ^†^	8.59 ± 5.91 *
Intercostal (*M. intercostales*)	6.75 ± 4.32	6.18 ± 3.25	0.802 ^†^	6.47 ± 3.77 *
Crus flexors (*Mm. flexores cruris*)	6.85 ± 4.78	7.45 ± 5.23	0.840 ^†^	7.16 ± 4.99 *
Forelimb flexors (*Mm. flexores antebrachii*)	6.89 ± 2.86	6.80 ± 3.43	0.959 ^†^	6.83 ± 2.96 *
Femoral biceps (*M. biceps femoris*)	6.04 ± 3.78	5.15 ± 3.46	0.680 ^†^	5.60 ± 3.54 *
Crus extensors (*Mm. extensores cruris*)	6.46 ± 3.01	6.04 ± 2.71	0.801 ^†^	6.23 ± 2.64 *
Back (*M*. *longissimus thoracis*)	4.14 ± 2.44	3.69 ± 2.10	0.735 ^†^	3.92 ± 2.26 *
*p* ^2^	-	-	-	<0.0001 ^§^
Tested muscles in total	9.79 ± 7.94 (7.27; 4.60–12.62)	9.96 ± 8.28 (7.49; 4.52–12.04)	0.994^‡^	13.84 ± 14.29 (8.15; 5.04–17.41)

SD—standard deviation; IQR—interquartile range. ^1^ The lpg value of each left muscle was compared with the lpg value of each right muscle. ^2^ The lpg value of diaphragm pillars was compared with the lpg value of the remaining 14 analyzed muscles (left and right combined). ^†^ Calculated using Student’s *t*-test. ^‡^ Calculated using Mann–Whitney *U*-test. ^§^ Calculated using one-way ANOVA followed by Tukey’s post hoc test. * The lpg value of a given muscle differs statistically from the lpg value of the diaphragm pillars.

**Table 2:** Distribution and intensity of *T. britovi* larvae infection (lpg) in muscles of pigs (n = 8) experimentally infected with 5000 muscle larvae of *T. britovi*.

Muscle/Muscle Group	Number of *Trichinella* Larvae/g Muscle (lpg)			
	Side		*p* ^1^	Left and Right (Combined)
	Left	Right		
	Mean ± SD (Median; IQR)	Mean ± SD (Median; IQR)		Mean ± SD (Median; IQR)
Diaphragm pillars (*Crura diaphragmatica*)	-	-	-	43.67 ± 16.57
Tongue (*Lingua*)	-	-	-	30.34 ± 11.42
Masseter (*M. masseter*)	14.42 ± 5.76	17.07 ± 4.52	0.324 ^†^	15.77 ± 4.63 *
Neck (*Mm. colli*)	14.39 ± 7.40	17.21 ± 5.65	0.406 ^†^	15.74 ± 5.47 *
Triceps brachii (*M. triceps brachii*)	12.68 ± 7.45	15.23 ± 7.93	0.517 ^†^	13.96 ± 6.36 *
Lateral and medial pterygoid (*M. pterygoideus lateralis et medialis*)	12.73 ± 7.58	14.78 ± 7.89	0.605 ^†^	13.82 ± 7.18 *
Sublumbar (*M. psoas major et minor*)	12.23 ± 4.98	12.37 ± 4.84	0.956 ^†^	12.30 ± 4.34 *
Abdomen (*Mm. abdominis*)	11.94 ± 7.83	11.67 ± 6.06	0.940 ^†^	11.81 ± 6.29 *
Forelimb extensors (*Mm. extensores antebrachii*)	7.86 ± 5.66	12.69 ± 6.66	0.140 ^†^	10.32 ± 6.00 *
Intercostal (*Mm. intercostales*)	8.94 ± 6.00	9.58 ± 3.61	0.800 ^†^	9.33 ± 4.33 *
Crus flexors (*Mm. flexores cruris*)	7.64 ± 6.16	10.88 ± 7.44	0.359 ^†^	9.19 ± 6.39 *
Forelimb flexors (*Mm. flexores antebrachii*)	7.47 ± 6.15	8.66 ± 5.56	0.692 ^†^	8.06 ± 5.69 *
Femoral biceps (*M. biceps femoris*)	6.64 ± 3.82	8.56 ± 5.02	0.404 ^†^	7.60 ± 3.90 *
Crus extensors (*Mm. extensores cruris*)	8.85 ± 4.98	6.33 ± 3.27	0.253 ^†^	7.50 ± 3.92 *
Back (*M. longissimus thoracis*)	6.42 ± 3.27	5.05 ± 2.36	0.355 ^†^	5.74 ± 2.65 *
*p* ^2^	-	-	-	<0.0001 ^§^
Tested muscles in total	10.17 ± 6.39 (8.21; 5.61–15.31)	11.54 ± 6.53 (10.29; 6.54–16.62)	0.105^‡^	14.34 ± 11.83 (11.16; 6.58–18.29)

SD—standard deviation; IQR—interquartile range. ^1^ The lpg value of each left muscle was compared with the lpg value of each right muscle. ^2^ The lpg value of diaphragm pillars was compared with the lpg value of the remaining 14 analyzed muscles (left and right combined). ^†^ Calculated using Student’s *t*-test. ^‡^ Calculated using Mann–Whitney *U*-test. ^§^ Calculated using Welch’s ANOVA followed by Games–Howell’s post hoc test. * The lpg value of a given muscle differs statistically from the lpg value of the diaphragm pillars.

**Table 3:** Comparison of the intensity of *T. britovi* ML infection (lpg) in individual muscles and the entire carcasses between groups of pigs infected with different doses of *T. britovi*.

Muscle/Muscle Group	Pigs Infected with 5000 ML of *T. britovi* (n = 8)	Pigs Infected with 3000 ML of *T. britovi* (n = 6)	*p* ^1^
	Numbers of *Trichinella* Larvae/g Muscle (lpg)		
	Median (IQR)		
Diaphragm pillars (*Crura diaphragmatica*)	49.32 (31.33–55.41)	38.38 (24.27–44.56)	0.561
Tongue (*Lingua*)	33.72 (18.97–39.97)	35.32 (23.48–37.04)	0.949
Masseter (*M. masseter*)	16.80 (11.82–19.37)	20.67 (17.24–21.30)	0.272
Neck (*Mm. colli*)	17.43 (10.74–20.27)	11.65 (7.94 –13.31)	0.401
Triceps brachii (*M. triceps brachii*)	11.79 (8.85–20.60)	8.75 (7.28–10.70)	0.220
Lateral and medial pterygoid (*M. pterygoideus lateralis et medialis*)	12.67 (6.87–20.77)	13.27 (6.67–17.41)	0.897
Sublumbar (*M. psoas major et minor*)	13.52 (8.85–15.20)	9.24 (8.05–9.91)	0.401
Abdomen (*Mm. abdominis*)	11.22 (6.80–14.19)	10.00 (6.73–13.24)	0.949
Forelimb extensors (*Mm. extensores antebrachii*)	8.58 (5.37–15.21)	6.92 (5.57–8.74)	0.561
Intercostal (*M. intercostales*)	8.71 (6.48–13.27)	4.88 (4.38–7.87)	0.272
Crus flexors (*Mm. flexores cruris*)	7.11 (4.07–13.59)	5.79 (3.66–8.12)	0.747
Forelimb flexors (*Mm. flexores antebrachii*)	7.21 (4.22–10.87)	5.51 (4.80–8.41)	0.846
Femoral biceps (*M. biceps femoris*)	7.35 (4.28–11.06)	4.65 (3.07–6.05)	0.366
Crus extensors (*Mm. extensores cruris*)	6.56 (4.13–10.94)	5.07 (4.31–7.61)	0.949
Back (*M. longissimus thoracis*)	5.85 (3.87–7.75)	3.08 (2.47–4.54)	0.220
15 tested muscles in total	11.16 (6.58–18.29)	8.15 (5.04–17.41)	0.106

IQR—interquartile range. ^1^ Calculated using Mann–Whitney *U*-test.

**Table 4:** Kinetics of IgG antibody response against ES antigens of *Trichinella* muscle larvae in the serum of pigs experimentally infected with 5000 ML of *Trichinella britovi* (n = 8). Analysis was performed with PrioCHECK^®^ *Trichinella* Ab, ID Screen^®^ *Trichinella* Indirect Multi-species, and Pigtype^®^ *Trichinella* Ab ELISAs.

Day before/after Infection	ELISA Test											
	PrioCHECK^®^ *Trichinella* Ab (ThermoFisher Scientific) ^1^				ID Screen^®^ *Trichinella* Indirect Multi-Species (ID Vet) ^2^				Pigtype^®^ *Trichinella* Ab (Qiagen) ^3^			
	OD			PP	OD			S/P%	OD			S/P
	Mean	±	SD	Mean	Mean	±	SD	Mean	Mean	±	SD	Mean
−4	0.065	±	0.004	2.258	0.097	±	0.012	2.660	0.104	±	0.031	0.041
3	0.073	±	0.009	2.526	0.104	±	0.020	3.148	0.121	±	0.031	0.056
6	0.089	±	0.056	3.089	0.171	±	0.191	7.507	0.197	±	0.082	0.121
9	0.096	±	0.076	3.319	0.179	±	0.189	7.436	0.221	±	0.088	0.142
13	0.102	±	0.065	3.548	0.172	±	0.158	7.621	0.250	±	0.085	0.166
15	0.123	±	0.068	4.288	0.171	±	0.134	7.511	0.251	±	0.108	0.173
17	0.149	±	0.083	5.199	0.192	±	0.142	8.829	0.237	±	0.089	0.166
20	0.117	±	0.066	4.126	0.169	±	0.128	7.033	0.231	±	0.124	0.160
24	0.113	±	0.061	3.979	0.167	±	0.110	6.958	0.234	±	0.108	0.163
27	0.157	±	0.117	5.512	0.215	±	0.169	10.093	0.295	±	0.104	0.194
29	0.245	±	0.275	8.602	0.324	±	0.292	17.405	0.376	±	0.267	0.256
36	**1.034 ^A^**	**±**	**0.801**	**36.321**	**1.017 ^A^**	**±**	**0.759**	**63.549**	**1.018 ^A^**	**±**	**0.761**	**0.802**
41	**1.733 ^B^**	**±**	**0.860**	**58.440**	**1.747 ^B^**	**±**	**0.696**	**111.099**	**2.020 ^B^**	**±**	**0.973**	**1.654**
45	**2.038 ^BC^**	**±**	**0.653**	**67.281**	**1.924 ^B^**	**±**	**0.389**	**122.164**	**2.339 ^B^**	**±**	**0.862**	**1.925**
51	**2.171 ^BC^**	**±**	**0.621**	**71.688**	**2.023 ^B^**	**±**	**0.226**	**128.623**	**2.375 ^B^**	**±**	**0.802**	**1.956**
55	**2.394 ^C^**	**±**	**0.528**	**79.028**	**2.016 ^B^**	**±**	**0.293**	**128.201**	**2.567 ^B^**	**±**	**0.694**	**2.119**
59	**2.435 ^C^**	**±**	**0.485**	**80.402**	**2.059 ^B^**	**±**	**0.284**	**131.025**	**2.323 ^B^**	**±**	**0.577**	**1.911**
62	**2.434 ^C^**	**±**	**0.481**	**80.816**	**2.145 ^B^**	**±**	**0.269**	**136.676**	**2.421 ^B^**	**±**	**0.544**	**1.995**

SD—standard deviation. ^1^ Cut-off value PP = 15% (PP ≥ 15%, positive result; PP < 15%, negative result). Positive test results are denoted by bold type. ^2^ Cut-off value S/P% = 60% (S/P% ≥ 60%, positive result; 50% ˂ S/P% ˂ 60%, doubtful result; S/P% ≤ 50%, negative result). Positive test results are denoted by bold type. ^3^ Cut-off value S/P = 0.3 (S/P ≥ 0.3, positive result; S/P < 0.3, negative result). Positive test results are denoted by bold type. The statistical analysis was performed using OD values. Different uppercase letters in the same columns indicate significant differences between the experimental periods within an experimental group in the particular ELISA procedure (*p* < 0.05; ANOVA with repeated measures following a post hoc Bonferroni test; these analyses were performed only for days that gave a positive result in the particular ELISA test). The average infection intensity of *T. britovi* in the 15 swine muscles analyzed (n = 8) was 14.34 larvae of *T. britovi*/g muscle.

**Table 5:** Performance of 3 ES-ELISA tests in the detection of anti-*Trichinella* IgG antibodies in pigs experimentally infected with 5000 ML of *T. britovi*: qualitative results.

Day before/after Infection	ELISA Test Result																							
	PrioCHECK^®^ *Trichinella* Ab (ThermoFisher Scientific)								ID Screen^®^ *Trichinella* Indirect Multi-Species (ID Vet)								Pigtype^®^ *Trichinella* Ab (Qiagen)							
	Pig no.								Pig no.								Pig no.							
	1	2	3	4	5	6	7	8	1	2	3	4	5	6	7	8	1	2	3	4	5	6	7	8
−4	−	−	−	−	−	−	−	−	−	−	−	−	−	−	−	−	−	−	−	−	−	−	−	−
3	−	−	−	−	−	−	−	−	−	−	−	−	−	−	−	−	−	−	−	−	−	−	−	−
6	−	−	−	−	−	−	−	−	−	−	−	−	−	−	−	−	−	−	−	−	−	−	−	−
9	−	−	−	−	−	−	−	−	−	−	−	−	−	−	−	−	−	−	−	−	−	−	−	−
13	−	−	−	−	−	−	−	−	−	−	−	−	−	−	−	−	−	−	−	−	−	−	−	−
15	−	−	−	−	−	−	−	−	−	−	−	−	−	−	−	−	−	−	−	−	−	−	−	−
17	−	−	−	−	−	−	−	−	−	−	−	−	−	−	−	−	−	−	−	−	−	−	−	−
20	−	−	−	−	−	−	−	−	−	−	−	−	−	−	−	−	−	−	−	−	−	−	−	−
24	−	−	−	−	−	−	−	−	−	−	−	−	−	−	−	−	−	−	−	−	−	−	−	−
27	−	−	−	−^1^	−	−	−	−	−	−	−	−	−	−	−	−	−	−	−	+	−	−	−	−
29	−	−	−	+	−	−	−	−	−	−	−	+	−	−	−	−	−^2^	−^2^	−	+	−	−	−	−
36	+	+	−	+	−^1^	+	−	+	+	+/−	−	+	−	+	−	+	+	+	−^2^	+	−	+	−	+
41	+	+	−^1^	+	+	+	+	+	+	+	+/−	+	+	+	+/−	+	+	+	+	+	+	+	+	+
45	+	+	+	+	+	+	+	+	+	+	+	+	+	+	+	+	+	+	+	+	+	+	+	+
51	+	+	+	+	+	+	+	+	+	+	+	+	+	+	+	+	+	+	+	+	+	+	+	+
55	+	+	+	+	+	+	+	+	+	+	+	+	+	+	+	+	+	+	+	+	+	+	+	+
59	+	+	+	+	+	+	+	+	+	+	+	+	+	+	+	+	+	+	+	+	+	+	+	+
62	+	+	+	+	+	+	+	+	+	+	+	+	+	+	+	+	+	+	+	+	+	+	+	+

−—negative test result; +—positive test result; +/−—doubtful results (according to the manufacturers’ protocols, doubtful results were generated only by the ID Screen^®^ *Trichinella* Indirect Multi-species test). ^1^ PP values in individual pigs reached a level close to the cut-off value (PP ≥ 15%, positive result; PP < 15%, negative result) and they were as follows: 13.057% (pig no. 3), 14.248% (pig no. 4), and 14.810% (pig no. 5). ^2^ S/P values in individual pigs reached a level close to the cut-off value (S/P ≥ 0.3, positive result; S/P < 0.3, negative result) and they were as follows: 0.287 (pig no. 1), 0.288 (pig no. 2), and 0.288 (pig no. 3). The mean intensity of *T. britovi* larvae infection (lpg) in 15 muscles of each pig was as follows: pig no. 1, 16.73 lpg; pig no. 2, 19.02 lpg; pig no. 3, 11.71 lpg; pig no. 4, 20.74 lpg; pig no. 5, 6.42 lpg; pig no. 6, 9.05 lpg; pig no. 7, 16.98 lpg; and pig no. 8, 14.08 lpg.

**Table 6:** Correlation between intensity of *T. britovi* ML infection (lpg) in muscles of pigs experimentally infected with 5000 ML of *T. britovi* and anti-*T. britovi* IgG level (OD) measured by three different commercial ELISA kits.

Muscle/Muscle Group	ELISA Test								
	PrioCHECK^®^ *Trichinella* Ab (ThermoFisher Scientific)			ID Screen^®^ *Trichinella* Indirect Multi-Species (ID Vet)			Pigtype^®^ *Trichinella* Ab (QIAGEN)		
	41 dpi	51 dpi	62 dpi	41 dpi	51 dpi	62 dpi	41 dpi	51 dpi	62 dpi
Diaphragm pillars (*Crura diaphragmatica*)	−0.276	−0.273	−0.699	−0.216	0.080	−0.142	−0.220	−0.070	−0.478
Tongue (*Lingua*)	−0.594	−0.654	−0.863	−0.482	−0.189	−0.089	−0.527	−0.420	−0.764
Masseter (*M. masseter*)	0.139	0.040	−0.382	0.101	−0.01	−0.380	0.164	0.228	−0.229
Neck (*Mm. colli*)	0.273	−0.080	−0.395	0.260	−0.202	−0.342	0.254	0.057	−0.272
Triceps brachii (*M. triceps brachii*)	0.093	−0.165	−0.419	−0.014	−0.541	−0.553	0.048	−0.104	−0.394
Lateral and medial pterygoid (*M. pterygoideus lateralis et medialis*)	0.245	0.058	−0.263	0.231	−0.258	−0.216	0.170	0.013	−0.192
Sublumbar (*M. psoas major et minor*)	0.388	0.019	−0.260	0.385	0.167	−0.198	0.434	0.277	−0.041
Abdomen (*Mm. abdominis*)	0.486	0.249	0.040	0.335	0.023	−0.436	0.502	0.408	0.144
Forearm extensors (*Mm. extensores antebrachii*)	0.706	0.411	0.214	0.622	0.120	−0.124	0.722 *	0.543	0.387
Intercostal (*Mm. intercostales*)	0.630	0.243	0.069	0.676	0.275	0.115	0.682	0.456	0.306
Crus flexors (*Mm. flexores cruris*)	0.482	0.248	0.102	0.364	−0.087	−0.016	0.490	0.303	0.313
Forearm flexors (*Mm. flexores antebrachii*)	0.547	0.250	0.078	0.386	−0.216	−0.290	0.516	0.285	0.199
Femoral biceps (*M. biceps femoris*)	0.485	0.228	−0.021	0.463	0.116	−0.185	0.536	0.456	0.146
Crus extensors (*Mm. extensores cruris*)	0.559	0.271	0.084	0.536	−0.006	0.080	0.541	0.297	0.272
Back (*M. Longissimus thoracis*)	0.328	−0.042	−0.323	0.287	−0.029	−0.193	0.332	0.109	−0.103

Data are Pearson’s *R* correlation coefficients. * Correlation is significant at the 0.05 level. Analysis was performed on the days on which at least one ELISA test classified all pigs as positive.

**Table 7:** Correlations between ELISA index values generated by PrioCHECK^®^ *Trichinella* Ab (PP), ID Screen^®^ *Trichinella* Indirect Multi-species (S/P%), and Pigtype^®^ *Trichinella* Ab (S/P) ELISAs in pigs experimentally infected with 5000 ML of *T. britovi*.

Pig no. (Serum Samples)	PrioCHECK^®^ *Trichinella* Ab/ ID Screen^®^ *Trichinella* Indirect Multi-Species	PrioCHECK^®^ *Trichinella* Ab/Pigtype^®^ *Trichinella* Ab	ID Screen^®^ *Trichinella* Indirect Multi-Species/Pigtype^®^ *Trichinella* Ab
1 (n = 18)	0.915 *	0.961 *	0.886 *
2 (n = 18)	0.950 *	0.884 *	0.953 *
3 (n = 18)	0.862 *	0.802 *	0.800 *
4 (n = 18)	0.928 *	0.930 *	0.938 *
5 (n = 18)	0.757 *	0.831 *	0.855 *
6 (n = 18)	0.963 *	0.895 *	0.907 *
7 (n = 18)	0.934 *	0.742 *	0.725 *
8 (n = 18)	0.777 *	0.764 *	0.847 *
Whole procedure (n = 144)	0.903 *	0.910 *	0.912 *

Data are Spearman’s *R* correlation coefficients; * Correlation is significant at the 0.05 level

## Data Availability

The data presented in this study are available on request from the corresponding author.

## References

[1] Murrell K.D., Pozio E. (2011). Worldwide occurrence and impact of human trichinellosis, 1986–2009. Emerg. Infect. Dis..

[2] Pozio E., Zarlenga D.S. (2005). Recent advances on the taxonomy, systematics and epidemiology of *Trichinella*. Int. J. Parasitol..

[3] Krivokapich S.J., Pozio E., Gatti G.M., Gonzalez Prous C.L., Ribicich M., Marucci G., La Rosa G., Confalonieri V. (2012). *Trichinella patagoniensis* n. sp. (Nematoda), a new encapsulated species infecting carnivorous mammals in South America. Int. J. Parasitol..

[4] Sharma R., Thompson P.C., Hoberg E.P., Scandrett W.B., Konecsni K., Harms N.J., Kukka P.M., Jung T.S., Elkin B., Mulders R. (2020). Hiding in plain sight: Discovery and phylogeography of a cryptic species of *Trichinella* (Nematoda: Trichinellidae) in wolverine (*Gulo gulo*). Int. J. Parasitol..

[5] Hurníková Z., Šnábel V., Pozio E., Reiterová K., Hrčková G., Halásová D., Dubinský P. (2005). First record of *Trichinella pseudospiralis* in the Slovak Republic found in domestic focus. Vet. Parasitol..

[6] Beck R., Beck A., Lučinger S., Florijančić T., Bošković I., Marinculić A. (2009). *Trichinella pseudospiralis* in pig from Croatia. Vet. Parasitol..

[7] Santrac V., Nedic D.N., Maric J., Nikolic S., Stevanovic O., Vasilev S., Cvetkovic J., Sofronic-Milosavljevic L. (2015). The first report of *Trichinella pseudospiralis* presence in domestic swine and *T. britovi* in wild boar in Bosnia and Herzegovina. Acta Parasitol..

[8] Pozio E., Rinaldi L., Marucci G., Musella V., Galati F., Cringoli G., Boireau P., La Rosa G. (2009). Hosts and habitats of *Trichinella spiralis* and *Trichinella britovi* in Europe. Int. J. Parasitol..

[9] Bilska-Zając E., Różycki M., Grądziel-Krukowska K., Bełcik A., Mizak I., Karamon J., Sroka J., Zdybel J., Cencek T. (2020). Diversity of *Trichinella* species in relation to the host species and geographical location. Vet. Parasitol..

[10] Bilska-Zając E., Różycki M., Chmurzyńska E., Marucci G., Cencek T., Karamon J., Bocian Ł. (2013). *Trichinella* species circulating in wild boars (*Sus scrofa*) populations in Poland. Int. J. Parasitol. Parasites Wildl..

[11] Pozio E., Hoberg E., La Rosa G., Zarlenga D.S. (2009). Molecular taxonomy, phylogeny and biogeography of nematodes belonging to the *Trichinella* genus. Infect. Genet. Evol..

[12] Cabaj W. (2006). Wild and domestic animals as permanent *Trichinella* reservoir in Poland. Wiad. Parazytol..

[13] Bilska-Zając E., Różycki M., Karamon J., Sroka J., Cencek T. (2019). The role of epidemiological investigations in the current epidemiology of trichinellosis in Poland. Życie Wet..

[14] Kapel C.M.O., Webster P., Lind P., Pozio E., Henriksen S.A., Murrell K.D., Nansen P. (1998). *Trichinella spiralis*, *T. britovi*, and *T. nativa*: Infectivity, larval distribution in muscle, and antibody response after experimental infection of pigs. Parasitol. Res..

[15] Malakauskas A., Kapel C.M.O., Webster P. (2001). Infectivity, persistence and serological response of nine *Trichinella* genotypes in rats. Parasite.

[16] Nöckler K., Serrano F.J., Boireau P., Kapel C.M.O., Pozio E. (2005). Experimental studies in pigs on *Trichinella* detection in different diagnostic matrices. Vet. Parasitol..

[17] Pozio E., Meriadli G., Licata E., Della Casa G., Fabiani M., Amati M., Cherchi S., Ramini M., Faeti V., Interisano M. (2020). Differences in larval survival and IgG response patterns in long-lasting infections by *Trichinella spiralis*, *Trichinella britovi* and *Trichinella pseudospiralis* in pigs. Parasit. Vectors.

[18] Gondek M., Knysz P., Pomorska-Mól M., Ziomek M., Bień-Kalinowska J. (2020). Acute phase protein pattern and antibody response in pigs experimentally infected with a moderate dose of *Trichinella spiralis*, *T. britovi*, and *T. pseudospiralis*. Vet. Parasitol..

[19] Gomez-Garcia V., Hernandez-Quero J., Rodriguez-Osorio M. (2003). Short report: Human infection with *Trichinella britovi* in Granada, Spain. Am. J. Trop. Med. Hyg..

[20] Akkoc N., Kuruuzum Z., Akar S., Yuce A., Onen F., Yapar N., Ozgenc O., Turk M., Ozdemir D., Avci M. (2009). A large-scale outbreak of trichinellosis caused by *Trichinella britovi* in Turkey. Zoonoses Public Health.

[21] Pavic S., Andric A., Sofronic-Milosavljevic L.J., Gnjatovic M., Mitić I., Vasilev S., Sparic R., Pavic A. (2020). *Trichinella britovi* outbreak: Epidemiological, clinical, and biological features. Med. Mal. Infect..

[22] Cabaj W., Moskwa B., Pastusiak K., Malczewski A. (2004). Trichinellosis in wild animals and domestic pigs in Poland. Med. Weter..

[23] Bień J., Moskwa B., Goździk K., Cybulska A., Kornacka A., Welc M., Popiołek M., Cabaj W. (2016). The occurrence of nematodes of the genus *Trichinella* in wolves (*Canis lupus*) from the Bieszczady Mountains and Augustowska Forest in Poland. Vet. Parasitol..

[24] Cybulska A., Kornacka A., Moskwa B. (2019). The occurrence and muscle distribution of *Trichinella britovi* in raccoon dogs (*Nyctereutes procyonoides*) in wildlife in the Głęboki Bród Forest District, Poland. Int. J. Parasitol. Parasites Wildl..

[25] Moskwa B., Goździk K., Bień J., Bogdaszewski M., Cabaj W. (2012). Molecular identification of *Trichinella britovi* in martens (*Martes martes*) and badgers (*Meles meles*); new host records in Poland. Acta Parasitol..

[26] General Veterinary Inspectorate Veterinary Statistical Reporting. 2015–2020. https://www.wetgiw.gov.pl/publikacje/rrw-sprawozdawczosc-statystyczna.

[27] (2015). Commission implementing regulation (EU) 2015/1375 of 10 August 2015 laying down specific rules on official controls for *Trichinella* in meat. Off. J. Eur. Union.

[28] (2015). Microbiology of the Food Chain—Detection of *Trichinella* Larvae in Meat by Artificial Digestion Method.

[29] Prost E.K., Nowakowski Z. (1990). Detectability of *Trichinella spiralis* in muscles by pooled-sample-digestion-method. Fleischwirtschaft.

[30] Franssen F.F.J., Fonville M., Takumi K., Vallée I., Grasset A., Koedam M.A., Wester P.W., Boireau P., van der Giesen J.W.B. (2011). Antibody response against *Trichinella spiralis* in experimentally infected rats is dose dependent. Vet. Res..

[31] Kapel C.M.O., Gamble H.R. (2000). Infectivity, persistence, and antibody response to domestic and sylvatic *Trichinella* spp. in experimentally infected pigs. Int. J. Parasitol..

[32] Gómez-Morales M.A., Ludovisi A., Amati M., Blaga R., Zivojinovic M., Ribicich M., Pozio E. (2012). A distinctive Western blot pattern to recognize *Trichinella* infections in humans and pigs. Int. J. Parasitol..

[33] Nöckler K., Voigt W.P., Protz D., Miko A., Ziedler K. (1995). Intravitale Diagnostik der Trichinellose beim Schwein mit dem indirekten ELISA. Berl. Münch. Tierärztl. Wschr..

[34] Gondek M., Bień J., Nowakowski Z. (2018). Use of ELISA and Western blot for serological detection of antibodies to E-S antigens of *Trichinella spiralis* muscle larvae in sera of swine experimentally infected with *Trichinella spiralis*. Vet. Immunol. Immunopathol..

[35] Grzelak S., Stachyra A., Stefaniak J., Mrówka K., Moskwa B., Bień-Kalinowska J. (2020). Immunoproteomic analysis of *Trichinella spiralis* and *Trichinella britovi* excretory-secretory muscle larvae proteins recognized by sera from humans infected with *Trichinella*. PLoS ONE.

[36] Zarlenga D.S., Chute M.B., Martin A., Kapel C.M.O. (1999). A multiplex PCR for unequivocal differentiation of all encapsulated and non-encapsulated genotypes of *Trichinella*. Int. J. Parasitol..

[37] Grzelak S., Moskwa B., Bień J. (2018). *Trichinella britovi* muscle larvae and adult worms: Stage-specific and common antigens detected by two-dimensional gel electrophoresis-based immunoblotting. Parasit. Vectors.

